# Inequalities in the benefits of national health insurance on financial protection from out-of-pocket payments and access to health services: cross-sectional evidence from Ghana

**DOI:** 10.1093/heapol/czz093

**Published:** 2019-09-20

**Authors:** Lucia Fiestas Navarrete, Simone Ghislandi, David Stuckler, Fabrizio Tediosi

**Affiliations:** 1 Department of Social and Political Science, Bocconi University, Via Roentgen 1, Milan, Italy; 2 Canadian Centre for Health Economics, 155 College Street, Toronto, ON, Canada; 3 Centre for Research on Health and Social Care Management, Bocconi University, Via Roentgen 1, Milan, Italy; 4 Carlo F. Dondena Centre for Research on Social Dynamics and Public Policy, Bocconi University, Via Roentgen 1, Milan, Italy; 5 Swiss Tropical and Public Health Institute, University of Basel, Socinstrasse 57, Basel, Switzerland

**Keywords:** *:* Universal health coverage, financial risk protection, utilization, out-of-pocket payments, health insurance, sociogeographic health inequalities, policy evaluation, Ghana

## Abstract

A central pillar of universal health coverage (UHC) is to achieve financial protection from catastrophic health expenditure. There are concerns, however, that national health insurance programmes with premiums may not benefit impoverished groups. In 2003, Ghana became the first sub-Saharan African country to introduce a National Health Insurance Scheme (NHIS) with progressively structured premium charges. In this study, we test the impact of being insured on utilization and financial risk protection compared with no enrolment, using the 2012–13 Ghana Living Standards Survey (*n* = 72 372). Consistent with previous studies, we observed that participating in health insurance significantly decreased the probability of unmet medical needs by 15 percentage points (p.p.) and that of incurring catastrophic out-of-pocket (OOP) health payments by 7 p.p. relative to no enrolment in the NHIS. Households living outside a 1-h radius to the nearest hospital had lower reductions in financial risk from excess OOP medical spending relative to households living closer (−5 p.p. vs −9 p.p.). We also find evidence that in Ghana, the scheme was highly pro-poor. Once insured, the poorest 40% of households experienced significantly larger improvements in medical utilization (18 p.p. vs. 8 p.p.) and substantively larger reductions in catastrophic OOP health expenditure (−10 p.p. vs. −6 p.p.) compared with that of the richest households. However, health insurance did not benefit vulnerable persons equally from financial risk. Once insured, poor, low-educated and self-employed households living far from hospitals had significantly lower reductions in catastrophic OOP medical spending compared with their counterparts living closer. Taken together, we show that enrolment in the NHIS is associated with improved financial protection but less so among geographically remote vulnerable groups. Efforts to boost not just insurance uptake but also health service delivery may be needed as a supplement for insurance schemes to accelerate progress towards UHC.

**Key Messages**

- In Ghana, participation in the National Health Insurance Scheme (NHIS) increased the probability of meeting medical needs and decreased the probability of incurring catastrophic out-of-pocket health payments.
- We reveal significant inequalities in the benefits derived from the NHIS across sociogeographic subgroups and find evidence that though the poorest benefit most from health insurance, these benefits are curtailed among vulnerable groups living outside a 1-h radius to the nearest hospital.
- Our study reveals the extent to which the social benefit of public health insurance derives from geographic accessibility to essential health facilities and highlights the socioeconomic groups for whom distance to care matters most.
- From a policy point of view, we show that improving the geographic availability of quality health services is as important as promoting enrolment in national health insurance schemes in order to boost progress towards universal coverage in low- and middle-income countries.

## Introduction

A strategic global health priority, universal health coverage (UHC), is widely recognized as the means to ensure that individuals do not suffer financial hardship when accessing quality health services (Hogan *et al.*, 2018). One major strategy is to expand health insurance coverage. Previously, studies have found that it can help to reduce the incidence of catastrophic health expenditure (Baicker *et al.*, 2013; Hu *et al.*, 2016) and out-of-pocket (OOP) health payments (King *et al.*, 2009; Chua and Sommers, 2014), as well as boost utilization of health services (Ghislandi *et al.*, 2015; Simon *et al.*, 2017), and population health outcomes (Sommers *et al.*, 2016, 2017). Yet, there are ongoing concerns that national health insurance programmes with premiums may not benefit high-risk and vulnerable groups, especially those who reside in peripheral and rural areas.

Ghana was the first sub-Saharan African (SSA) country to introduce a National Health Insurance Scheme (NHIS). Previous studies have assessed the catastrophic and impoverishment effects of OOP health payments prior to the introduction of the NHIS in Ghana (Akazili *et al.*, 2017a,b). They find that 10.7% of Ghanaian households spent >10% of their non-food consumption expenditure on OOP health payments (Akazili *et al.*, 2017a). Consistent with the international literature, a study by Fenny *et al.* (2018) using data from three Ghanaian districts showed that insured individuals were more likely to seek care for the treatment of malaria, while a study conducted in the Eastern and Central regions found that insurance reduced OOP payments and protected households against impoverishment (Aryeetey *et al.*, 2016).

Although there is a consensus that health insurance can improve utilization and financial risk protection among the insured, the literature offers conflicting evidence on the protective effect of insurance among high-risk beneficiaries. Based on a large randomized assessment of ‘Seguro Popular’, the Mexican health insurance programme, King *et al.* (2009) found that the poorest beneficiaries of insurance experienced greater reductions in catastrophic health expenditure. In contrast, a study by Lu *et al.* (2012) evaluating the impact of ‘Mutuelles’, the Rwandan community-based health insurance programme, found that the poorest beneficiaries had the lowest rates of utilization and highest rates of catastrophic expenditure. Moreover, a recent study by Grogger *et al.* (2015) found that beneficiaries living in areas with access to single-nucleus health facilities experience significantly lower reductions in catastrophic expenditure compared with rural-dwelling beneficiaries with access to larger facilities. Though Grogger *et al.*’s findings regard beneficiaries with access to differently staffed facilities, they offer insights into the potentially moderating effect of distance to care on the relationship between health insurance and financial risk protection.

A growing body of work has recognized the effect of distance and travel time to health facilities on utilization. Karra *et al.* (2016) pooled data from 21 low- and middle-income countries (LMICs) to estimate associations among facility distance, child mortality and utilization. Their findings show that the children living within 2, 3 and 5 km of a facility have 8%, 16%,and 25% higher odds of neonatal mortality, respectively, compared with that of the children living within 1 km distance. Masters *et al.* (2013) investigated the effect of travel time on the likelihood of in-facility delivery (IFD) among rural households in Ghana and found that a 1 h increase in travel time reduced the odds of IFD by 24%. While the accruing literature reveals important associations between travel time and utilization, there is a lacuna of studies investigating the potentially moderating role of travel time in the relationship between insurance, utilization and catastrophic expenditure. Moreover, considering the large heterogeneity of populations with limited access to healthcare facilities, a limitation of prior work is an inability to disaggregate findings by social position and test the hypothesis of differential benefit among geographically remote disenfranchized groups.

To address these gaps, we draw on the 2012–13 Ghana Living Standards Survey data (*n* = 72 371) and examine the impact of the first NHIS in SSA in its first 10 years of implementation. We stratify population subgroups based on travel time to the nearest hospital and household socioeconomic characteristics to evaluate the effect of health insurance on financial risk protection and utilization among high-risk and vulnerable beneficiaries with and without limited geographic accessibility to care. We use probit models with region fixed effects, which were further tested using propensity score matching (PSM) and instrumental variable (IV) estimation methods to address potential selection bias into insurance. Using this sample, we test the hypothesis that the poorest benefit more from national health insurance schemes, but that this is attenuated for beneficiaries living in remote settings.

### Ghana’s NHIS

Established in 2003, the NHIS sought to eliminate user fees and eradicate the financial barriers created by earlier reforms. In the pre-NHIS policy period, OOP payments contributed 48% of the total health expenditure (Leive and Xu, 2008). The current NHIS offers free access to a package of diagnostic, inpatient and outpatient services covering 95% of conditions afflicting Ghanaians (NHIS, 2018). The scheme is characterized by a ‘mandatory-voluntary’ ‘mode of participation’ that effectively creates a three-tier enrolment structure whereby (1) formal workers are automatically covered through deductible social contributions, (2) informal workers are covered voluntarily through annual premium payments and (3) vulnerable persons are exempted from paying premiums altogether.

Premiums range from 7.20 to 48 Ghana Cedis (GHS; USD 1.60–10.60) per adult annually, varying according to region of residence. Vulnerable groups that qualify for exemptions include children under 18, adults over 70, pregnant women, individuals with disabilities and indigents. Every member must pay an initial processing fee towards a membership card (GHS 8, c.a. USD 1.82) and a yearly renewal fee (GHS 5, c.a. USD 1.14). Individuals who are not registered in the NHIS are obliged to make OOP payments every time they access health services, which may result in financial hardship.

Mixed participation generates a differential ‘basis for benefit entitlement’: contributory for formal workers, discretionary for informal workers and non-contributory for vulnerable persons. Though coverage varies widely as a result, Ghana’s scheme type is not uncommon among LMICs experimenting with health financing reforms as part of broader UHC strategies (Tangcharoensathien *et al.*, 2011).

## Materials and methods

### Source of data

We use data from the sixth Ghana Living Standards Survey (GLSS-6). The details have been described elsewhere [Ghana Statistical Service (GSS), 2014] but briefly, GLSS-6 is a nationwide representative household survey conducted by the Ghana Statistical Service in 2012–13. A two-stage stratified random sampling framework was employed at both regional and national levels. In the first stage, 1200 enumeration areas (i.e. clusters) were sampled across 10 geographic regions with weighted probabilities proportional to population size. In the second stage, 15 households were randomly selected from each cluster. Thus, covering a nationally representative sample of 72 372 individuals living within 16 772 households across 1200 clusters. We restricted the study sample to individuals who were either enrolled in the NHIS (treatment group) or did not have any form of insurance (control group).

### Outcome measures

Catastrophic expenditure is a binary outcome variable indicating whether OOP health payments absorbed an excessive share of the household budget. OOP health payments consist of annual household level spending on both inpatient and outpatient services and all other reported spending directly related to the receipt of health services. We express OOP health payments as a ratio of total household non-food consumption (Wagstaff *et al.*, 2018), which is obtained by deducting total annual food consumption (F) from each household’s total annual real consumption${\;(C}_{h}):{\mathrm{OOP}}_{h}/\left(C_{h}-F_{h}\right)$. Catastrophic expenditure corresponds to OOP health payments that absorb >10% of household non-food consumption: $x<{\mathrm{OOP}}_{h}/\left(C_{h}-F_{h}\right)<1$, where x=0.1. Utilization is a binary outcome variable indicating whether an individual used medical services if she were ill or injured in the previous 2 weeks of the survey. Although our utilization outcome variable operates at the individual level, the financial risk protection outcome variable operates at the household level. This reflects the fact that: (1) expenditure is an intra-household, rather than individual, decision and (2) GLSS-6 reports expenditure data only at the household level.

### Independent variables

We created a binary variable ‘NHIS’ to represent an individual’s participation in the NHIS, where 1= insured and 0, otherwise. The sociodemographic variables contained in the medical utilization analysis include age and gender of the respondent, gender, education and employment status of the household head, household size, household consumption expenditure and rural residence. We included a binary variable to indicate whether an individual lives in a household with at least one elderly member, as well as a dummy variable indicating whether an individual lives in a household located outside a 1-h travel radius to the nearest hospital. A radio ownership dummy variable was built to detect the effect of public health education, often accessed via radio programming. We used two dummy variables to indicate whether an individual who self-reported illness or injury in the 2 weeks prior to the survey was forced to stop her usual activities due to the ailment’s severity and whether an individual suffered from any kind of disability. We included 10 regional dummy variables to control for heterogeneity of unobserved health systems-related characteristics across regions. Due to the household-level nature of the financial risk protection analysis, we use insured households as the ‘NHIS’ treatment group, whereby household insurance status derives from that of the household head.

### Statistical models

We use probit models with region fixed effects to estimate the impact of health insurance on the probability that an individual uses medical care when ill or injured, and the probability that an individual lives in a household that incurs catastrophic OOP health expenditure (Equation 1). Each model postulates that utilization ${(m}_{1})$ and financial risk protection ${(m}_{2})$ are functions of insurance status $c_{1}{\mathrm{NHIS}}_{i}$, in addition to sociodemographic, household and geographic characteristics:

(1) $$Y_{\mathrm{ihr}}^{m_{1,\;2}}=\;\alpha_{1}+c_{1}{\mathrm{NHIS}}_{i}+X_{i}^{'}\beta_{1}+W_{\mathrm{ih}}^{'}\delta_{1}+Z_{\mathrm{ihr}}^{'}\zeta_{1}+\varepsilon_{1i\mathrm{hr}}$$

where individual-level (i) variables are represented by the vector β, household-level (h) variables by the vector δ and region-level (r) dummy variables by the vector ζ.

### Evaluating potential effect differences

To examine the effect of health insurance on utilization, we restricted our study sample to individuals who reported being sick or injured in the 2 weeks prior to the survey (*n* = 10 311), whereas to study financial risk protection, we restricted our sample to individuals whose households made any OOP health payments in the year of the survey (*n* = 25 971). To evaluate differences in the effect of health insurance across vulnerable subgroups, we disaggregated our sample across levels of household socioeconomic characteristics. These include household consumption expenditure, education and employment status of the household head. Across each subgroup, we tested whether travel time to care (within *vs.* outside a 1-h radius to the nearest hospital) influences the effect of insurance on utilization and financial risk protection.

We use travel time to care since it implicitly encompasses not only distance but also difficulty of travel and may better reflect the decision-making process to utilize services (Masters *et al.*, 2013). We select hospitals as the single site from which to derive travel time because they tend to offer a comprehensive array of services available through the NHIS (e.g. diagnostic, inpatient and outpatient). The median time travelled to the nearest hospital in our sample is 60 min (IQR: 60) and the mean 73 (SD: 69.4). Hence, we proceed with a median-split to categorize subpopulations within and outside a 1-h travel time. Aligned with the relevant literature, previous studies based in SSA countries confirm that travel times to health facilities of at least 1 h present a sufficient barrier to access services (Okwaraji *et al.*, 2012; Masters *et al.*, 2013).

### Identification strategy

A key methodological challenge facing our study is the requirement that the individual decision to enrol in health insurance be uncorrelated with observable and unobservable determinants of utilization and health expenditure. This assumption is challenging, as insurance status is likely to incorporate an *ex* *ante* need for medical care, with the consequent problem of selection bias. The importance of testing and accounting for potential endogeneity of insurance participation in models explaining variability in health service use and catastrophic expenditure has been investigated widely (Hellinger and Wong, 2000; Liang *et al.*, 2004).

Drawing from the approach used by Lu *et al.* (2012), we constructed a measure of cluster NHIS insurance prevalence rate and used it as an IV to approximate an exogenous source of variation in insurance participation. Our data are composed of 1200 clusters, each representing a demarcated geographic area that consists of 15 households. The NHIS prevalence rate for an individual i living in cluster k equals the number of insured persons in cluster k minus the insurance status of the same individual divided by the total number of persons in the cluster. The assumptions that individuals living in geographic clusters characterized by high insurance rates are more likely to be insured (relevance) and that cluster insurance rate affects neither an individual’s decision to use medical services nor a household’s decision to spend on health directly (exclusion) are reasonable and discussed in Lu *et al.* (2012).

We postulate that a correlation between the endogenous regressor and our instrument is possible for different reasons. For example: (1) clusters of enrolled individuals might arise because residents in some geographical areas share higher quality of medical services, and (2) individuals living in a geographic area with a higher concentration of insured individuals may be influenced by the enrolment behaviour of their peers (DiMaggio and Garip, 2012). The peer-effect claim is supported by a recent study, which revealed that presenting health insurance information to informal groups had a larger effect on retention and trust in the insurance scheme than full premium subsidies (Chemin, 2018).

We included cluster insurance rate in first-stage probit regressions and obtained the predicted probabilities of NHIS participation for each individual:

(2) $${\mathrm{NHIS}}_{i}=\;\alpha_{2}+c_{2}{\mathrm{cluster}\;\mathrm{rate}}_{i}+X_{i}^{'}\beta_{2}+W_{\mathrm{ih}}^{'}\delta_{2}+Z_{\mathrm{ihr}}^{'}\zeta_{2}+\varepsilon_{2i\mathrm{hr}}$$

which were used to estimate the effect of health insurance on utilization and financial risk protection in the respective second-stage IV regressions:

(3) $$Y_{\mathrm{ihr}}^{m_{1,\;2}}=\;\alpha_{3}+{c_{3}N\hat{\mathrm{HI}}S}_{i}+X_{i}^{'}\beta_{3}+W_{\mathrm{ih}}^{'}\delta_{3}+Z_{\mathrm{ihr}}^{'}\zeta_{3}+\varepsilon_{3i\mathrm{hr}}$$

To mitigate possible selection bias due to observable characteristics, a PSM estimator was calculated, using NHIS-affiliated individuals as the treatment group (Imbens, 2004). We used the nearest neighbour (NN) matching without replacement approach and restricted matching within a calliper of 0.0001 to avoid matching by a neighbour very far from the insured individual but with the closest propensity score.

We matched treated and control individuals based on covariates that may influence selection into insurance. For the utilization outcome equation, we matched individuals based on (1) demographic (i.e. age, gender), (2) individual-level medical need (i.e. illness severity), (3) head-of-household (i.e. education, employment status), (4) household (i.e. consumption expenditure, size) and (5) geographic (i.e. rural residence and travel time) characteristics. For the catastrophic expenditure outcome equation, we matched individuals based on (1) head-of-household (i.e. age, gender, education, employment status), (2) household (i.e. consumption expenditure, size), (3) household-level medical need (i.e. presence of elderly members, disabled members, ill members) and (4) geographic (i.e. rural residence, travel time) characteristics. When conditioning on these covariates, the observed outcomes of uninsured units can be reasonably used to estimate the counterfactual outcome of insured units in the case of no treatment.

**Figure 1:**
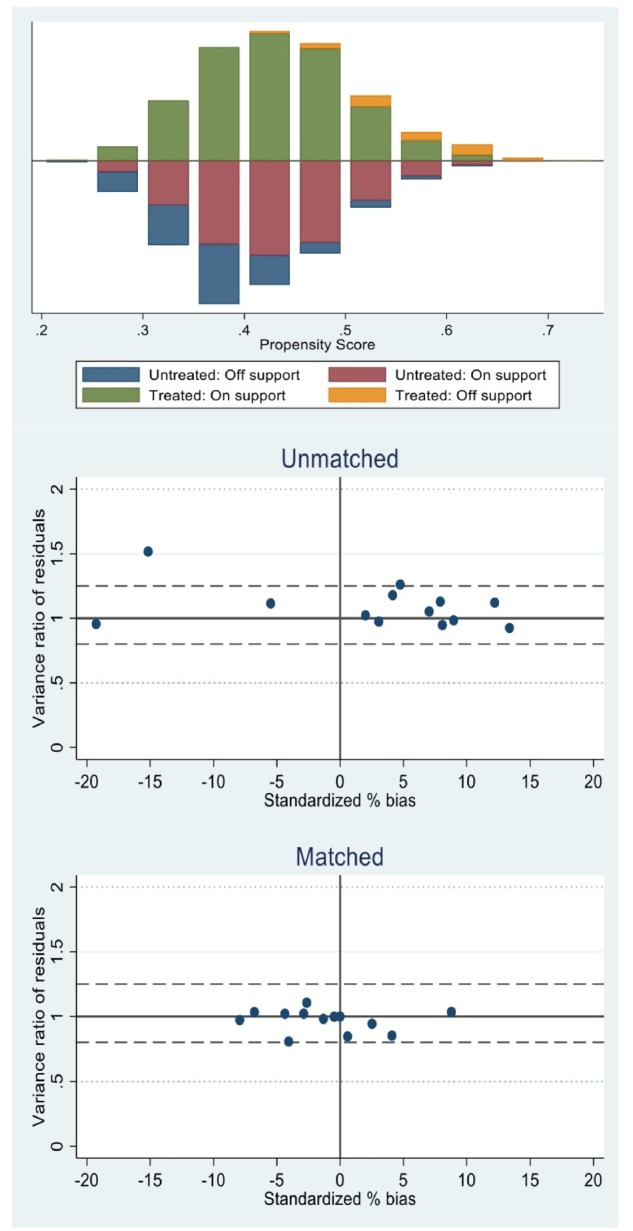
Distribution of propensity scores using nearest neighbour matching for medical utilization across treatment and comparison groups and representation of standardized bias between matched and unmatched samples, Ghana 2012–13.

**Figure 2:**
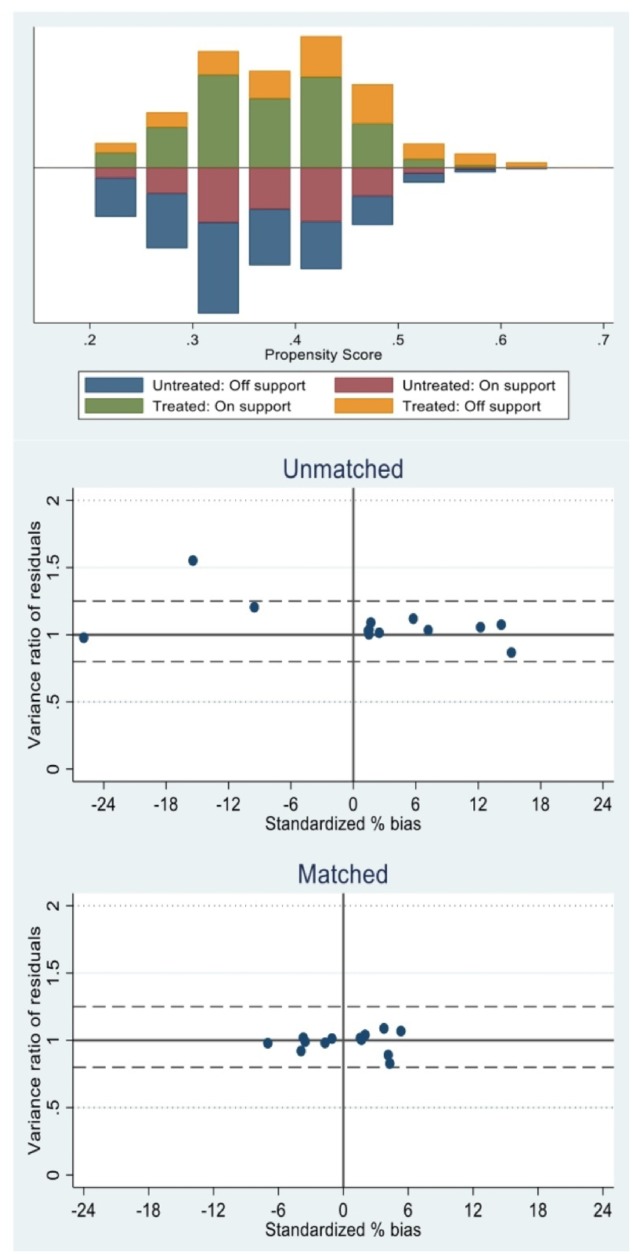
Distribution of propensity scores using nearest neighbour matching for financial risk protection across treatment and comparison groups and representation of standardized bias between matched and unmatched samples, Ghana 2012–13.

Standardized differences and *t*-tests for the covariates used to satisfy the balancing property offer evidence that the propensity scores were properly identified (see Supplementary Tables S1 and S2). These tables report, separately for the two outcomes, the mean characteristics by insurance status. Differences between the insured and uninsured groups are arguably small and become even smaller after matching. These are the subsets of treated and control subjects that are effectively used in the estimation of the causal effect of interest throughout the matched probit specifications (without and with IV). Common support for each model can be assessed by examining the distribution of propensity scores across groups (Figures 1 and 2).

## Results

Table 1 presents descriptive statistics for insured and uninsured groups in our sample. About 36% of individuals were insured by the NHIS. Among the 45 405 uninsured individuals, 16% were insured in the past but had failed to renew their annual NHIS membership, whereas the remaining 84% had never been insured. The most frequently reported reason for never having registered for health insurance (63%) and for failing to renew the NHIS membership (38%) was having ‘No money’. As it regards enrolment, 67% of insured individuals became NHIS members by paying a premium, whereas 31% qualified for a premium exemption. The mean premium payment was GHS 7.74. Moreover, within premium exempted groups, insured individuals were a persistent minority: 38% of children under 18, 48% of adults over 70, 46% of pregnant women and 37% of individuals living with disabilities were insured.

**Table 1:** Descriptive statistics, Ghana 2012–13

	Uninsured		NHIS Insured
	*N* (%)		*N* (%)
		
Individuals	45 405 (63.68)		25 894 (36.32)
Households	11 292 (67.44)		5452 (32.56)
Age categories			
Under 5	5844 (12.87)		3584 (13.84)
5–18	16 057 (35.36)		9659 (37.30)
19–44	16 001 (35.24)		7827 (30.23)
45–74	6697 (14.75)		4039 (15.60)
75 and older	806 (1.78)		785 (3.03)
Female	22 720 (50.04)		13 998 (54.06)
Education of household head			
No schooling	15 081 (33.24)		8537 (32.99)
Up to primary	11 551 (25.46)		6157 (23.79)
More than primary	18 733 (41.29)		11 187 (43.22)
Household head is self-employed	34 562 (80.28)		19 473 (78.97)
Expenditure quintiles			
Poorest	14 001 (30.84)		7347 (28.37)
Poorer	9305 (20.40)		5626 (21.73)
Middle	8070 (17.77)		4835 (18.67)
Richer	7323 (16.13)		4246 (16.40)
Richest	6706 (14.77)		3840 (14.83)
Health need and medical care utilization (2 weeks)			
Illness or injury	6149 (13.56)		4162 (16.10)
Stopped activities due to severity	3692 (59.99)		2697 (64.61)
Sought care due to illness or injury	3699 (60.16)		3131 (75.23)
OOP health expenditure by quintile			
All households	6391 (56.60)		2993 (54.90)
Poor	1234 (19.31)		484 (16.17)
Poorer	1197 (18.73)		554 (18.51)
Middle	1224 (19.15)		604 (20.18)
Richer	1256 (19.65)		615 (20.55)
Richest	1480 (23.16)		736 (24.59)
All households	552 (4.62)		232 (4.26)
Poorest	145 (1.28)		43 (0.79)
Poorer	132 (1.17)		43 (0.79)
Middle	108 (0.96)		51 (0.94)
Richer	91 (0.81)		50 (0.92)
Richest	76 (1.19)		45 (0.83)
Hospital >1 h^a^	12 545 (46.64)		5503 (33.50)
Rural residence	27 919 (61.49)		16 239 (62.71)

a Merged from Section 42 of the GLSS 6 Community questionnaire, which collected information on distance to health facilities using a reduced sample of 44 056 individuals within 643 clusters.

### Medical care utilization

Table 2 presents probit regression results generated from the unmatched data, PSM data and PSM data with IV for utilization analyses in the sample of individuals that reported illness or injury 2 weeks prior to the survey. Results from the first-stage IV-probit regression are shown in Column (3), providing strong evidence that the cluster insurance rate significantly predicts participation in the NHIS. Findings on the effect of the NHIS on utilization are positive, sizeable and significant across specifications: individuals insured by the NHIS are more likely to use medical services when needed compared with their uninsured counterparts after controlling for other factors.

**Table 2:** Utilization results using probit models with unmatched data, propensity score matched data (PSM) and matched data with instrumental variable (PSM-IV), Ghana 2012–13

	Medical care when ill or injured			
	(1)	(2)	(3)	(4)
	Unmatched	PSM	First-stage PSM-IV	PSM-IV
NHIS	0.43***	0.43***	.	0.22***
	(0.36–0.50)	(0.35–0.51)		(0.05–0.39)
Cluster insurance rate			0.98***	
			(0.93–1.02)	
Age categories				
Under 5	(Reference)			
				
5–18	−0.30***	−0.30***	−0.03	−0.30***
	(−0.40 to −0.20)	(−0.41 to −0.18)	(−0.06 to 0.01)	(−0.41 to −0.19)
19–44	−0.30***	−0.28***	−0.10***	−0.30***
	(−0.40 to −0.19)	(−0.39 to −0.16)	(−0.13 to −0.06)	(−0.41 to −0.18)
45–74	−0.34***	−0.34***	−0.03	−0.34***
	(−0.45 to −0.23)	(−0.46 to −0.21)	(−0.06 to 0.01)	(−0.46 to −0.21)
75 and older	−0.35***	−0.28***	0.06*	−0.27**
	(−0.54 to −0.16)	(−0.49 to −0.07)	(−0.00 to 0.13)	(−0.48 to −0.05)
Female	0.06*	0.05	0.02	0.04
	(−0.00 to 0.13)	(−0.10 to 0.19)	(−0.02 to 0.07)	(−0.10 to 0.19)
Female household head	−0.05	−0.08	0.00	−0.08
	(−0.14 to 0.03)	(−0.19 to 0.03)	(−0.03 to 0.04)	(−0.18 to 0.03)
Education of household head				
No schooling	(Reference)			
				
Up to primary	0.07	0.08*	0.02	0.09*
	(−0.02 to 0.15)	(−0.01 to 0.18)	(−0.02 to 0.05)	(−0.01 to 0.18)
More than primary	0.08	0.04	0.05***	0.05
	(−0.02 to 0.17)	(−0.08 to 0.15)	(0.02–0.09)	(−0.06 to 0.16)
Household head is self-employed	0.07	−0.02	0.01	−0.01
	(−0.04 to 0.19)	(−0.15 to 0.11)	(−0.04 to 0.05)	(−0.15 to 0.12)
Expenditure quintiles				
Poorest	(Reference)			
				
Poorer	0.16***	0.15***	0.03	0.16***
	(0.07–0.25)	(0.04–0.26)	(−0.01 to 0.06)	(0.05–0.27)
Middle	0.21***	0.22***	0.03	0.22***
	(0.10–0.31)	(0.08–0.36)	(−0.01 to 0.07)	(0.08–0.36)
Richer	0.27***	0.26***	0.03	0.26***
	(0.15–0.40)	(0.08–0.44)	(−0.03 to 0.09)	(0.08–0.44)
Richest	0.29***	0.25**	0.05	0.26**
	(0.14–0.44)	(0.02–0.48)	(−0.01 to 0.12)	(0.03–0.49)
Household size	0.01	0.01	0.00	0.00
	(−0.00 to 0.02)	(−0.01 to 0.03)	(−0.01 to 0.01)	(−0.02 to 0.02)
Severity of illness or injury	0.45***	0.45***	0.01	0.45***
	(0.38–0.52)	(0.31–0.59)	(−0.03 to 0.05)	(0.30–0.59)
Hospital >1 h	−0.08**	−0.08	0.07*	−0.07
	(−0.15 to −0.01)	(−0.36 to 0.19)	(−0.01 to 0.16)	(−0.34 to 0.20)
Rural residence	−0.20**	−0.27	0.13***	−0.23
	(−0.36 to −0.04)	(−0.62 to 0.09)	(0.03–0.23)	(−0.58 to 0.12)
				
Observations	6307	4920	4920	4920
Controls and Region FE included	Yes	Yes	Yes	Yes
Wald test *P*-value			<0.001	

Robust 95% confidence intervals are given in parentheses. Controls include 10 region dummies, disability, cohabitation with elderly members and radio ownership.

*** *P* < 0.01,

** *P* < 0.05,

* *P* < 0.1.

### Financial risk protection

Table 3 presents probit regression results for the financial risk protection analyses generated from the unmatched data, PSM data and PSM data with IV. Column (3) shows the results from the first-stage IV-probit regression, which instruments health insurance with cluster insurance rate and offers strong evidence that the instrument significantly predicts participation in the NHIS. Findings are consistently negative and significant across specifications: after controlling for covariates, individuals enrolled in the NHIS are significantly less likely to live in households that incur catastrophic health expenditure.

**Table 3:** Financial risk protection results using probit models with unmatched data, propensity score matched data (PSM) and matched data with instrumental variable (PSM-IV), Ghana 2012 − 13

	OOP payment exceeds 10% of non-food consumption			
	(1)	(2)	(3)	(4)
	Unmatched	PSM	First-stage PSM-IV	PSM-IV
NHIS	−0.14***	−0.12***		−0.47***
	(−0.19 to −0.09)	(−0.19 to −0.05)		(−0.66 to −0.29)
Cluster insurance rate			0.79***	
			(0.76–0.82)	
Age of household head	0.00*	0.00	−0.00***	0.00
	(−0.00 to 0.00)	(−0.00 to 0.01)	(−0.00 to −0.00)	(−0.00 to −0.01)
Female household head	0.16***	0.30***	0.00	0.29***
	(0.10–0.23)	(0.19–0.40)	(−0.03 to 0.03)	(0.19–0.40)
Education of household head				
No schooling	(Reference)			
** **				
Up to primary	−0.05	0.04	0.00	0.04
	(−0.10 to 0.01)	(−0.06 to 0.14)	(−0.02 to 0.03)	(−0.06 to 0.14)
More than primary	−0.06*	0.09	0.04**	0.10
	(−0.13 to 0.00)	(−0.07 to 0.24)	(0.00–0.08)	(−0.06 to 0.26)
Household head is self-employed	0.01	0.08	0.07***	0.10
	(−0.08 to 0.10)	(−0.09 to 0.25)	(0.03–0.10)	(−0.06 to 0.27)
Expenditure quintiles				
Poorest	(Reference)			
Poorer	−0.07**	−0.03	0.02*	−0.02
	(−0.13 to −0.01)	(−0.12 to 0.07)	(−0.00 to 0.04)	(−0.12 to 0.07)
Middle	−0.28***	−0.25***	0.03*	−0.25***
	(−0.36 to −0.21)	(−0.38 to −0.12)	(−0.00 to 0.06)	(−0.38 to −0.11)
Richer	−0.33***	−0.34***	0.01	−0.34***
	(−0.42 to −0.24)	(−0.52 to −0.16)	(−0.03 to 0.05)	(−0.52 to −0.16)
Richest	−0.63***	−0.48***	−0.04	−0.49***
	(−0.75 to −0.50)	(−0.71 to −0.26)	(−0.09 to 0.01)	(−0.72 to −0.27)
Household size	−0.07***	−0.05***	−0.00	−0.05***
	(−0.08 to −0.06)	(−0.07 to −0.04)	(−0.01 to 0.00)	(−0.07 to −0.04)
Hospital > 1hr	0.03	0.04	0.15***	0.09
	(−0.02 to 0.08)	(−0.35 to 0.43)	(0.06–0.24)	(−0.30 to 0.48)
Rural residence	0.20***	−0.03	0.18***	0.02
** **	(0.07–0.33)	(−0.35 to 0.29)	(0.10–0.26)	(−0.31 to 0.34)
Observations	25 971	12 684	12 684	12 684
Catastrophic OOP observations	2089	936	936	936
Non-Catastrophic OOP observations	23 882	11 748	11 748	11 748
Controls and region FE included	YES	YES	YES	YES
Wald test *P*-value			<0.001	

Robust 95% confidence intervals are given in parentheses. Controls include 10 region dummies, disability, disease severity, cohabitation with elderly members and radio ownership.

*** *P* < 0.01,

** *P* < 0.05,

* *P* < 0.1.

The NHIS coefficient in Tables 2 and 3 remains stable across models, changing slightly with the IV estimation. Since we have no prior regarding the size and direction of coefficient changes when the IV is implemented, these results show that the impact of the NHIS is robust and in the expected direction. The fact that the NHIS coefficient on utilization is smaller in the PSM-IV analysis implicitly confirms that the instrument addresses selection bias into insurance. Assuming that the IV approach overcomes the bias of naïve estimators, we suggest that coefficients associated with the PSM-IV specifications represent the effect that we are actually interested in—that of health insurance on a sample of individuals who comply with the assignment to the treatment given by cluster rate. Hence, we use PSM-IV specifications to compute local average treatment effect estimates when disentangling main effects into subgroup estimates.

A common objection to the classic catastrophic expenditure definition employed here is that it ignores important differences in the budget capacity of poor and non-poor households. To test the robustness of our results, we used Wagstaff and Eozenou’s (2014) unified financial risk protection methodology, yielding unique outcome variables relevant to population groups above and below the poverty line (see Supplementary Figure S1). The comprehensive rationale and implementation of the method can be found in the original article (Wagstaff and Eozenou, 2014). Our results are robust to the use of different outcome variables. Table 4 shows that enrolment in the NHIS significantly reduces financial hardship resulting from OOP health payments among families living above and below the poverty line.

**Table 4:** Financial risk protection results using outcome variables derived from the unified financial risk protection methodology, Ghana 2012–13

	Poor households^a^			Non-poor households^b^								
* *	Log OOP immiseration burden on household discretionary consumption			OOP payments absorb >25% of household discretionary consumption			OOP payments leave household consumption below 110% of the poverty line		
	(1)	(2)	(3)	(4)	(5)	(6)	(7)	(8)	(9)
	Unmatched	PSM	PSM-IV	Unmatched	PSM	PSM-IV	Unmatched	PSM	PSM-IV
									
NHIS	−0.14***	−0.06	−0.31**	−0.14***	−0.13**	−0.57***	0.07**	−0.17***	−0.33**
	(−0.19 to −0.08)	(−0.13 to 0.02)	(−0.56 to −0.07)	(−0.23 to −0.06)	(−0.26 to −0.01)	(−0.91 to −0.23)	(0.00–0.13)	(−0.27 to −0.07)	(−0.64 to −0.02)
									
Cluster insurance (first stage)			0.68***			0.74***			0.72***
			(0.63–0.73)			(0.68–0.79)			(0.66–0.78)
									
Observations	13 645	5780	5780	12 080	5038	5038	10 542	4161	4161
*R*-squared	0.30	0.29	0.29				** **	** **	** **
*F*-statistic *P*-value			<0.001						
Wald test *P*-value						0.01			0.30

Robust 95% confidence intervals are given in parentheses. Columns (1) through (3) show linear regression models. Columns (4) through (9) show probit models. All specifications include controls and region fixed effects. Controls include 10 region dummies, age, gender, education and employment status of household head, household consumption expenditure quintiles, household size, distance to nearest hospital, rural residence, disability, disease severity, cohabitation with elderly members and radio ownership.

a Households that were below the poverty line prior to incurring OOP health spending.

b Households that were above the poverty line prior to incurring OOP health spending.

*** *P* < 0.01,

** *P* < 0.05,

* *P* < 0.1.

### Heterogeneity by proximity to care

Table 5 presents the effect estimates of health insurance on utilization and financial risk protection. Our results show that enrolment in the NHIS increases the probability of meeting medical needs by 15 percentage points (p.p.) while decreasing the probability of incurring catastrophic OOP health payments by 7 p.p. relative to no enrolment. When disaggregating the population based on proximity to care, we observe that the effect of insurance on improved utilization is larger among insured individuals living within a 1-h travel time to the nearest hospital (17 p.p. increase) than for individuals living farther than 1 h away (14 p.p. increase). We also observe that the effect of health insurance on improved financial risk protection is larger among insured individuals living within a 1-h radius to the nearest hospital (9 p.p. decrease in catastrophic expenditure) than for insured individuals living farther (5 p.p. decrease). Overall, the effects of health insurance on improved utilization and financial risk protection are most pronounced among insured individuals living within 1-h travel time to a hospital.

**Table 5:** Effect estimates of health insurance on medical utilization and financial risk protection by distance to nearest hospital using IV-probit models and propensity score matched datasets, Ghana 2012–13

	Local average treatment effect	
* *	Medical care utilization when ill or injured	OOP payment exceeds 10% of non -food consumption
	(1)	(2)
	PSM-IV probit	PSM-IV probit
		
All individuals	**0.15** ***	**−0.07** ***
	(0.13–0.18)	(−0.10 to −0.03)
	4920	12 684
		
Individuals living *within* 1-h radius to nearest hospital	**0.17** ***	**−0.09** ***
	(0.13–0.20)	(−0.13 to −0.05)
	3003	7803
		
Individuals living *outside* 1-h radius to nearest hospital	**0.14** ***	−0.05**
	(0.09–0.18)	(−0.09 to −0.004)
	1917	4881
		
(T-statistic *P*-value)	(0.15)	(0.07)
	** **	** **

Numbers in bold are estimated effects. The 95% confidence intervals are given in parentheses. Last number in each cell is the sample size.

*** *P* < 0.01,

** *P* < 0.05,

* *P* < 0.1.

### Differences in utilization by socioeconomic factors and proximity to care

Table 6 presents effect estimates of health insurance on the probability of utilization across different socioeconomic subgroups and disaggregated by proximity to care. Enrolment in the NHIS has a positive, sizable and statistically significant effect on medical service use across socioeconomic subgroups relative to no enrolment. The effect of health insurance on improved utilization is significantly larger among the poorest 40% of the population (18 p.p. increase), compared with that of the richest 40% (8 p.p. increase; *P* = 0.003). When we disaggregate socioeconomic groups based on proximity to care, we find that vulnerable groups (i.e. individuals living in poorer, lower educated and self-employed households) benefit consistently less from health insurance when living outside a 1-h radius from the nearest hospital.

**Table 6:** Bootstrapped local average treatment effect (LATE) estimates of health insurance on medical care utilization using IV-probit models and propensity score matched datasets, Ghana 2012 − 13

	Household consumption expenditure		Education of household head		Employment of household head	
	(1)	(2)	(3)	(4)	(5)	(6)
	Poorest 40%	Richest 40%	Up to primary	>Primary	Self-employed	Employed
All individuals	**0.18** ***	**0.08** **	**0.17** ***	**0.13** ***	**0.15** ***	**0.16** ***
	(0.14–0.21)	(0.02–0.14)	(0.14–0.19)	(0.07–0.19)	(0.12–0.18)	(0.07–0.25)
	2921	1054	3309	1627	4381	555
(T-statistic *P*-value)	(0.003)		(0.11)		(0.41)	
						
	Distance and poverty		Distance and education		Distance and employment	
Individuals living *within* 1-h radius to nearest hospital	**0.20** ***	**0.09** **	**0.18** ***	**0.15** ***	**0.17** ***	**0.14** ***
	(0.16–0.25)	(0.02–0.15)	(0.13–0.22)	(0.10–0.21)	(0.13–0.21)	(0.04–0.25)
	1589	761	1863	1132	2573	422
Individuals living *outside* 1-h radius to nearest hospital	**0.15** ***	**0.10**	**0.16** ***	**0.08** *	**0.13** ***	**0.42**
	(0.09–0.21)	(−0.04 to 0.23)	(0.11–0.20)	(−0.01 to 0.17)	(0.08–0.17)	(−0.08 to 0.92)
	1332	293	1446	495	1808	119
(T-statistic *P*-value)^a^	(0.07)	(0.44)	(0.25)	(0.09)	(0.08)	(0.053)

Numbers in bold are estimated effects. The 95% confidence intervals are given in parentheses. Last number in each cell is the sample size.

a *P*-values from T-statistics correspond to effect differences between rows 2 and 3.

*** *P* < 0.01,

** *P* < 0.05,

* *P* < 0.1.

### Differences in financial risk protection by socioeconomic factors and proximity to care

Table 7 presents the effect estimates of health insurance on the probability of catastrophic OOP health expenditure across socioeconomic subgroups and disaggregated by proximity to care. Overall, enrolment in the NHIS has a negative, sizable and statistically significant effect on financial risk due to catastrophic health expenditure across socioeconomic subgroups relative to no enrolment. The effect of health insurance on improved financial risk protection is larger among the poorest households (10 p.p. decrease in catastrophic expenditure), compared with that of the richest (6 p.p. decrease; *P* < 0.10). We observe larger reductions of catastrophic health expenditure among households headed by members with higher compared with that of the lower education (14 p.p. vs 3 p.p.; *P* < 0.000) and among households headed by employed, compared with that of the self-employed members (16 p.p. vs 6 p.p.; *P* = 0.04). When we disaggregate socioeconomic groups based on proximity to care, we consistently find that vulnerable groups who live farther than 1 h away from the nearest hospital benefit significantly less from the financial protection afforded by health insurance.

**Table 7:** Bootstrapped local average treatment effect (LATE) estimates of health insurance on catastrophic out-of-pocket health expenditure using IV-probit models and propensity score matched datasets, Ghana 2012–13

	Household consumption expenditure		Education of household head		Employment of household head	
	(1)	(2)	(3)	(4)	(5)	(6)
	Poorest 40%	Richest 40%	Up to primary	> Primary	Self-employed	Employed
All individuals	−0.10***	−0.06**	−0.03*	−0.14***	−0.06***	−0.16**
	(−0.14 to −0.07)	(−0.10 to −0.01)	(−0.07 to 0.003)	(−0.18 to −0.09)	(−0.09 to −0.03)	(−0.32 to −0.01)
	7624	2336	8603	4081	11 471	927
(T-statistic *P*-value)	(0.10)		(<0.001)		(0.04)	
						
	Distance and poverty		Distance and education		Distance and employment	
Individuals living *within* 1-h radius to nearest hospital	−0.13***	−**0.04**	−0.07***	−0.16***	−0.09***	−**0.13**
	(−0.18 to −0.07)	(−0.08 to 0.01)	(−0.11 to −0.02)	(−0.22 to −0.10)	(−0.13 to −0.04)	(−0.35 to 0.10)
	4465	1603	5145	2630	6881	851
Individuals living *outside* 1-h radius to nearest hospital	−0.07**	−0.24**	−**0.02**	−0.19***	−**0.04**	−0.26***
	(−0.12 to −0.01)	(−0.44 to −0.04)	(−0.07 to 0.04)	(−0.31 to −0.08)	(−0.09 to 0.02)	(−0.39 to −0.13)
	3128	474	3407	1182	4519	362
(T-statistic *P*-value)^a^	(0.06)	(0.002)	(0.07)	(0.29)	(0.08)	(0.24)

Numbers in bold are estimated effects. The 95% confidence intervals are given in parentheses. Last number in each cell is the sample size.

a *P*-values from T-statistics correspond to effect differences between rows 2 and 3.

*** *P* < 0.01,

** *P* < 0.05,

* *P* < 0.1.

### Robustness checks

We conducted a series of robustness and sensitivity tests on our PSM models by comparing relative effects across three alternative matching methods. In addition to NN without replacement, we applied kernel, radius and Mahalanobis matching. We verified the covariate balance graphically across matching procedures by comparing the standardized bias in matched and unmatched samples (see Supplementary Figures S2 and S3). In addition, we used two balancing tests for each alternative method: standardized differences and *t*-tests (see Supplementary Tables S3–S8) and estimated average treatment effects on the treated (ATT) for each outcome variable obtained from the four matching methods. Table 8 shows that the ATT estimates for the two outcomes do not change significantly between matching methods.

**Table 8:** Average treatment effects on the treated (ATT) across matching methods, Ghana 2012–13

	Medical utilization				Catastrophic health expenditure			
	N treated	N control	ATT	95% CI	N treated	N control	ATT	95% CI
Nearest neighbour	2468	2476	0.155***	(0.131–0.180)	6342	6532	−0.022***	(−0.026 to −0.018)
							
Radius	2617	3605	0.155***	(0.130–0.179)	9024	13 881	−0.023***	(−0.033 to −0.014)
							
Kernel	2666	3641	0.157***	(0.134–0.181)	9615	16 356	−0.026***	(−0.033 to −0.019)
							
Mahalanobis	2666	3641	0.145***	(0.114–0.175)	9615	16 356	−0.006	(−0.013 to 0.002)
								

CI, confidence intervals.

*** *P* < 0.01,

** *P* < 0.05,

* *P* < 0.1.

We also conducted simulation-based sensitivity analyses allowing us to assess whether the ATT estimates are robust to failures of unconfoundedness. All sensitivity analyses convey robustness of the matching estimate with respect to reasonable failures of the conditional independence assumption (see Supplementary Tables S9–S12). The comprehensive rationale and implementation of the method can be found in the original article (Ichino *et al.*, 2008).

## Conclusion

Detecting the conditions under which national health insurance systems offer protection to the insured and identifying the least protected beneficiaries is an important, albeit largely under-investigated area of research. Our findings show that participation in the NHIS increased the probability of meeting medical needs and decreased the probability of incurring catastrophic OOP health payments relative to no enrolment. We reveal significant effect differences across socioeconomic subgroups and find evidence that the poorest benefit most from health insurance, though these benefits are significantly curtailed among geographically remote vulnerable groups.

We consistently find that poorer beneficiaries living outside a 1-h travel time to the nearest hospital benefit significantly less from the financially protective effect of health insurance. The fact that higher travel times are associated with utilization and financial protection penalties among vulnerable beneficiaries reveals an insightful decision-making mechanism. Poorer, less educated and precariously employed geographically remote households tend to forgo care, despite being insured, due to the time, difficulty and/or costs associated with reaching a health facility. For households faced by the disincentive of living far from a hospital, being enrolled in insurance is not a sufficiently effective incentive to utilize services even with the expectation of free care upon arrival.

We show that being enrolled in the NHIS may still not be sufficient to ensure financial risk protection and access to health services among the most disenfranchized sociogeographic subgroups. They highlight that insurance schemes are unlikely to safeguard financial protection from catastrophic expenditure if higher-level healthcare facilities are not geographically accessible. Our findings are in line with a recent analysis of the Community-based Health Planning and Services initiative in Ghana, which underlined the importance of bridging geographical access to healthcare as a prerequisite to delivering on the promise of universal coverage (Assan *et al.*, 2018).

Our findings are consistent with recent work by Grogger *et al.* (2015) who showed that ‘Seguro Popular’ provided greater financial protection in areas proximate to larger health facilities. In addition to confirming these findings, the most novel contribution of our paper is to unveil the differential effects of health insurance by distance to care and socioeconomic characteristics. In doing so, we sought to draw more convincing conclusions regarding the benefits of health insurance as experienced by families with distinctive *a priori* degrees of vulnerability. Our results are also aligned with those obtained by previous studies on Ghana (Akazili *et al.*, 2017a,b) and elsewhere (van Doorslaer *et al.*, 2007), which voiced the inherent challenge of providing financial protection to the most vulnerable beneficiaries. Taken together, our findings confirm that improving the geographic availability of quality health services is as important as promoting enrolment in national health insurance schemes in order to boost progress towards UHC.

Moreover, the fact that households headed by less-educated members benefit less from the financially protective effect of health insurance indicates that navigating and securing the benefits of a national health insurance product is dependent upon the education level of beneficiaries. This partially reflects Hart’s (1971) inverse care logic, explaining why beneficiaries with low education levels and reasonably poor understanding of health insurance would be less able to leverage insurance claims.

To ensure that the benefits of health insurance be experienced equitably across sociogeographic groups, UHC-driven policies should be enhanced with parallel improvements in transport infrastructure and focused expansion of the current hospital network to poorly serviced geographic areas. Our findings suggest that travel time is at least one of the decision-making components compelling insured individuals to seek or forgo needed healthcare. As such, we recommend the implementation of targeted health education interventions aiming to incentivize prompt care-seeking behaviour among geographically remote vulnerable groups. Our findings also indicate shortcomings concerning the implementation of policies meant to protect vulnerable people. In Ghana, vulnerable groups are exempted from paying enrolment premiums, however, the implementation of these policies is challenging. There may be important underlying conflicts between healthcare providers facing budget constraints and reimbursement uncertainty, and policies seeking to broaden access to care among vulnerable beneficiaries. Thus, implementation inefficiencies may be part of the explanation as to why some of the most vulnerable NHIS enrolees are least protected from financial hardship.

These implications extend well-beyond Ghana, as other SSA countries with similar fiscal constraints are experimenting with hybrid health insurance schemes alike. Among them, Rwanda and Ethiopia have exemptions built-in their health financing structures aiming to target destitute groups. Our findings suggest that, although exemptions are part of the way forward, closer attention should be paid to long-term investments in road quality, supply network expansion and health education policies. Indeed, by targeting the junction of social, economic and geographic vulnerability, policymakers may be better able to identify a burdened high-risk group that is not yet benefitting from health insurance equitably despite the presence of well-intentioned exemptions.

These findings should be viewed in light of the following limitations. First, although the comprehensive objectives that our work seeks to examine include access to promotive, preventive, curative, rehabilitative and palliative health services, we are able to assess the impact of health insurance on medical utilization focusing on curative care only. Second, though we consider UHC not as an end in and of itself but the means towards better health outcomes, our study assesses the effect of health insurance on improved health service use. Although there is a reason to believe that access to care leads to improved health outcomes, we do not directly measure the effect of the NHIS on these outcomes. Third, due to the data availability our study measures utilization 2 weeks prior to the survey and as such, offers a partial picture of utilization and a lower bound estimate of annual health service use. Fourth, the cross-sectional nature of our data has allowed us to capture annual OOP health expenditure at the time of the survey, which we have found to be sufficient to affect household financial well-being. However, it is possible that households incur recurrent catastrophic health expenditures, whose consequences may be more detrimental, and for which longitudinal data are needed.

Overall, this study supports the UHC objective of the Ghanaian NHIS and offers valuable lessons to other LMICs seeking to broaden access to quality healthcare while lessening reliance on OOP payments. To our knowledge, our study is the first to investigate the effect of health insurance on utilization and financial risk protection across socioeconomic characteristics based on travel time to care. Our findings point to the need for developing more effective approaches to include vulnerable sociogeographic groups in nascent national health insurance systems and to ensure that they benefit equitably from utilization and financial protection. Finally, in an effort to identify the conditions under which health insurance offers protection to vulnerable beneficiaries, our study offers a novel contribution to the literature from a policy point of view. We reveal the extent to which the social benefit of health insurance derives from geographic accessibility to essential health facilities and highlight the socioeconomic groups for whom distance to care matters most.

*Ethical approval.* No ethical approval was required for this study.

## Supplementary Material

czz093_Supplementary_Data
