# Supplementary material for: Inequalities in the benefits of national health insurance on financial protection from out-of-pocket payments and access to health services: cross-sectional evidence from Ghana
Source: Health Policy Plan. 2019 Sep 20;34(9):694–705. doi: 10.1093/heapol/czz093 (PMC6880330; doi:10.1093/heapol/czz093)
Supplement: czz093_Supplementary_Data [file czz093_supplementary_data.zip › czz093-Suppl_data/Supplementary Table 12.docx]

| **Table S12.** Sensitivity analysis of the propensity score for catastrophic expenditure: characterizing ‘killer’ confounders, Ghana 2012-2013 | | | | | | |
| --- | --- | --- | --- | --- | --- | --- |
|  | *s* = 0.1 | *s* = 0.2 | *s* = 0.3 | *s* = 0.4 | *s* = 0.5 | *s* = 0.6 |
|  | 𝛬 ∈ [1.4, 1.8] | 𝛬 ∈ [2.1, 2.7] | 𝛬 ∈ [3.2, 4.3] | 𝛬 ∈ [5.2, 9.6] | 𝛬 ∈ [9.8, 27.6] | 𝛬 ∈ [32.8, 40.1] |
|  |  |  |  |  |  |  |
| d = 0.1 𝛤 ∈ [1.5] | -0.03 | -0.03 | -0.03 | -0.03 | -0.03 | -0.03 |
|  | (-0.04 – -0.01) | (-0.05 – -0.01) | (-0.05 – -0.01) | (-0.05 – -0.01) | (-0.05 – -0.01) | (-0.06 – -0.01) |
| d = 0.2 𝛤 ∈ [2.2, 2.6] | -0.03 | -0.03 | -0.03 | -0.04 | -0.04 | -0.04 |
|  | (-0.04 – -0.01) | (-0.05 – -0.02) | (-0.05 – -0.02) | (-0.05 – -0.02) | (-0.06 – -0.02) | (-0.06 – -0.02) |
| d = 0.3 𝛤 ∈ [3.6, 3.7] | -0.03 | -0.04 | -0.04 | -0.05 | -0.05 | -0.06 |
|  | (-0.05 – -0.02) | (-0.05 – -0.02) | (-0.06 – -0.03) | (-0.07 – -0.03) | (-0.08 – -0.03) | (-0.08 – -0.04) |
| d = 0.4 𝛤 ∈ [6.9, 7.0] | -0.04 | -0.04 | -0.05 | -0.06 | -0.07 | -0.08 |
|  | (-0.05 – -0.02) | (-0.06 – -0.03) | (-0.07 – -0.04) | (-0.08 – -0.04) | (-0.09 – -0.05) | (-0.11 – -0.06) |
| d = 0.5 𝛤 ∈ [23.3, 23.7] | -0.06 | -0.08 | -0.11 | -0.14 | -0.16 | -0.19 |
|  | (-0.08 – -0.04) | (-0.10 – -0.07) | (-0.13 – -0.09) | (-0.16 – -0.11) | (-0.19 – -0.13) | (-0.22 – -0.15) |
| d = 0.6 𝛤 ∈ [ . ] | -0.17 | -0.26 | -0.35 | -0.45 | -0.54 | -0.63 |
|  | (-0.18 – -0.15) | (-0.28 – -0.24) | (-0.37 – -0.33) | (-0.47 – -0.42) | (-0.57 – -0.51) | (-0.67 – -0.60) |
|  |  |  |  |  |  |  |
| The differences $d=p_{01}-p_{00}$ and s$=p_{1\cdot}-p_{0\cdot}$ capture the outcome effect of *U* in the absence of treatment and the effect of *U* on the selection into treatment, respectively. *d* and *s* uniquely define the parameters $p_{ij}$, with $i,j \epsilon\left\{ 0,1 \right\}.$ The simulated ATTs associated to the corresponding differences *d* (in rows) and *s* (in columns) are shown in each cell (95% Confidence Intervals in parentheses). Each ATT is averaged over 100 iterations. 𝛤 denotes the average estimated odds ratio of *U* in the logit model of $\Pr(Y=1\vert T=0,U,W)$. 𝛬 is the average estimated odds ratio of *U* in the logit model of $\Pr(T=1,U,W)$. The baseline estimate without confounder is -0.02 (95% CI: -0.03 – -0.01). | | | | | | |
