# Supplementary material for: Inequalities in the benefits of national health insurance on financial protection from out-of-pocket payments and access to health services: cross-sectional evidence from Ghana
Source: Health Policy Plan. 2019 Sep 20;34(9):694–705. doi: 10.1093/heapol/czz093 (PMC6880330; doi:10.1093/heapol/czz093)
Supplement: czz093_Supplementary_Data [file czz093_supplementary_data.zip › czz093-Suppl_data/Supplementary Table 1.docx]

| **Table S1.** Test of the balancing property of the propensity score for medical care utilization, Ghana 2012-2013 | | | | | | | | |
| --- | --- | --- | --- | --- | --- | --- | --- | --- |
|  |  |  |  |  |  |  |  |  |
| Sample | Mean | |  |  | % reduction |  |  |  |
|  | Insured | Uninsured |  | % bias | bias |  | p>\|t\| |  |
| Age (years) |  |  |  |  |  |  |  |  |
| Unmatched | 28.32 | 27.32 |  | 4.2 |  |  | 0.10 |  |
| Matched | 27.90 | 28.53 |  | -2.6 | 36.4 |  | 0.39 |  |
| Gender |  |  |  |  |  |  |  |  |
| Unmatched | 0.57 | 0.52 |  | 9 |  |  | <0.001 |  |
| Matched | 0.55 | 0.56 |  | -0.5 | 94.7 |  | 0.88 |  |
| Gender of head |  |  |  |  |  |  |  |  |
| Unmatched | 0.22 | 0.21 |  | 2 |  |  | 0.43 |  |
| Matched | 0.22 | 0.22 |  | -1.3 | 33.9 |  | 0.66 |  |
| Education of head |  |  |  |  |  |  |  |  |
| Unmatched | 1.00 | 0.94 |  | 7 |  |  | 0.01 |  |
| Matched | 0.98 | 1.00 |  | -2.9 | 59.1 |  | 0.34 |  |
| Head self-employed |  |  |  |  |  |  |  |  |
| Unmatched | 0.88 | 0.89 |  | -5.5 |  |  | 0.03 |  |
| Matched | 0.89 | 0.88 |  | 2.5 | 54.1 |  | 0.40 |  |
| Rural |  |  |  |  |  |  |  |  |
| Unmatched | 1.92 | 1.95 |  | -15.2 |  |  | <0.001 |  |
| Matched | 1.95 | 1.94 |  | 4.1 | 73 |  | 0.16 |  |
| Household size |  |  |  |  |  |  |  |  |
| Unmatched | 5.96 | 5.85 |  | 3.1 |  |  | 0.23 |  |
| Matched | 5.97 | 5.95 |  | 0.6 | 80.9 |  | 0.85 |  |
| Elderly household member | |  |  |  |  |  |  |  |
| Unmatched | 0.22 | 0.19 |  | 7.9 |  |  | <0.001 |  |
| Matched | 0.21 | 0.21 |  | 0 | 99.9 |  | 1.00 |  |
| Expenditure (quintiles) |  |  |  |  |  |  |  |  |
| Unmatched | 2.41 | 2.25 |  | 12.2 |  |  | <0.001 |  |
| Matched | 2.35 | 2.44 |  | -6.8 | 44.4 |  | 0.03 |  |
| Hospital > 1hr |  |  |  |  |  |  |  |  |
| Unmatched | 0.37 | 0.46 |  | -19.3 |  |  | <0.001 |  |
| Matched | 0.39 | 0.35 |  | 8.8 | 54.4 |  | <0.001 |  |
| Radio ownership |  |  |  |  |  |  |  |  |
| Unmatched | 0.70 | 0.64 |  | 13.4 |  |  | <0.001 |  |
| Matched | 0.69 | 0.72 |  | -7.9 | 40.6 |  | 0.01 |  |
| Severity of illness or injury | |  |  |  |  |  |  |  |
| Unmatched | 0.67 | 0.63 |  | 8.1 |  |  | <0.001 |  |
| Matched | 0.66 | 0.68 |  | -4.4 | 45.9 |  | 0.15 |  |
| Disability |  |  |  |  |  |  |  |  |
| Unmatched | 0.04 | 0.03 |  | 4.7 |  |  | 0.06 |  |
| Matched | 0.03 | 0.04 |  | -4.1 | 14.3 |  | 0.17 |  |
|  |  |  |  |  |  |  |  |  |
