# Supplementary material for: Inequalities in the benefits of national health insurance on financial protection from out-of-pocket payments and access to health services: cross-sectional evidence from Ghana
Source: Health Policy Plan. 2019 Sep 20;34(9):694–705. doi: 10.1093/heapol/czz093 (PMC6880330; doi:10.1093/heapol/czz093)
Supplement: czz093_Supplementary_Data [file czz093_supplementary_data.zip › czz093-Suppl_data/Supplementary Table 2.docx]

| **Table S2.** Test of the balancing property of the propensity score for catastrophic out-of-pocket health expenditure, Ghana 2012-2013 | | | | | | | |
| --- | --- | --- | --- | --- | --- | --- | --- |
| Sample | Mean | |  |  | % reduction |  | |
|  | Insured | Uninsured |  | % bias | bias |  | p>\|t\| |
| Age of head (years) |  |  |  |  |  |  |  |
| Unmatched | 48.93 | 47.86 |  | 7.2 |  |  | <0.001 |
| Matched | 48.10 | 48.67 |  | -3.9 | 45.7 |  | 0.05 |
| Gender of head |  |  |  |  |  |  |  |
| Unmatched | 0.17 | 0.16 |  | 1.4 |  |  | 0.27 |
| Matched | 0.17 | 0.18 |  | -1.7 | -19.3 |  | 0.40 |
| Education of head |  |  |  |  |  |  |  |
| Unmatched | 0.99 | 0.89 |  | 12.2 |  |  | <0.001 |
| Matched | 0.97 | 0.95 |  | 2 | 83.5 |  | 0.31 |
| Head self-employed |  |  |  |  |  |  |  |
| Unmatched | 0.88 | 0.91 |  | -9.5 |  |  | <0.001 |
| Matched | 0.91 | 0.89 |  | 4.1 | 56.5 |  | 0.03 |
| Rural |  |  |  |  |  |  |  |
| Unmatched | 1.92 | 1.96 |  | -15.4 |  |  | <0.001 |
| Matched | 1.96 | 1.95 |  | 4.3 | 72.2 |  | 0.01 |
| Household size |  |  |  |  |  |  |  |
| Unmatched | 6.85 | 6.79 |  | 1.7 |  |  | 0.19 |
| Matched | 6.62 | 6.57 |  | 1.6 | 7.7 |  | 0.42 |
| Elderly household member | |  |  |  |  |  |  |
| Unmatched | 0.19 | 0.17 |  | 5.8 |  |  | <0.001 |
| Matched | 0.19 | 0.18 |  | 1.7 | 70.9 |  | 0.40 |
| Expenditure (quintiles) |  |  |  |  |  |  |  |
| Unmatched | 2.41 | 2.23 |  | 14.2 |  |  | <0.001 |
| Matched | 2.31 | 2.40 |  | -7 | 50.9 |  | <0.001 |
| Hospital > 1hr |  |  |  |  |  |  |  |
| Unmatched | 0.34 | 0.47 |  | -25.9 |  |  | <0.001 |
| Matched | 0.39 | 0.36 |  | 5.3 | 79.5 |  | 0.01 |
| Radio ownership |  |  |  |  |  |  |  |
| Unmatched | 0.74 | 0.67 |  | 15.2 |  |  | <0.001 |
| Matched | 0.73 | 0.73 |  | -1.1 | 93 |  | 0.59 |
| Household member sick or injured | |  |  |  |  |  |  |
| Unmatched | 0.57 | 0.56 |  | 1.5 |  |  | 0.24 |
| Matched | 0.57 | 0.59 |  | -3.7 | -146.9 |  | 0.06 |
| Household member severely sick or injured | |  |  |  |  |  |  |
| Unmatched | 0.43 | 0.42 |  | 2.5 |  |  | 0.05 |
| Matched | 0.44 | 0.45 |  | -3.5 | -41.9 |  | 0.08 |
| Household member with disability | |  |  |  |  |  |  |
| Unmatched | 0.10 | 0.09 |  | 1.5 |  |  | 0.24 |
| Matched | 0.10 | 0.09 |  | 3.8 | -147.2 |  | 0.06 |
|  |  |  |  |  |  |  |  |
|  |  |  |  |  |  |  |  |
