# Supplementary material for: Inequalities in the benefits of national health insurance on financial protection from out-of-pocket payments and access to health services: cross-sectional evidence from Ghana
Source: Health Policy Plan. 2019 Sep 20;34(9):694–705. doi: 10.1093/heapol/czz093 (PMC6880330; doi:10.1093/heapol/czz093)
Supplement: czz093_Supplementary_Data [file czz093_supplementary_data.zip › czz093-Suppl_data/Supplementary Table 5.docx]

| **Table S5.** Tests of balancing properties for medical care utilization using Mahalanobis matching, Ghana 2012-2013 | | | | | | | | |
| --- | --- | --- | --- | --- | --- | --- | --- | --- |
| Sample | Mean | |  |  | % reduction |  |  |  |
|  | Insured | Uninsured |  | % bias | bias |  | p>\|t\| |  |
| Age (years) |  |  |  |  |  |  |  |  |
| Unmatched | 28.32 | 27.32 |  | 4.2 |  |  | 0.10 |  |
| Matched | 28.32 | 27.28 |  | 4.3 | -3.8 |  | 0.12 |  |
| Gender |  |  |  |  |  |  |  |  |
| Unmatched | 0.57 | 0.52 |  | 9 |  |  | <0.001 |  |
| Matched | 0.57 | 0.57 |  | -1.1 | 88.2 |  | 0.70 |  |
| Gender of head |  |  |  |  |  |  |  |  |
| Unmatched | 0.22 | 0.21 |  | 2 |  |  | 0.43 |  |
| Matched | 0.22 | 0.22 |  | 1.2 | 40.9 |  | 0.67 |  |
| Education of head |  |  |  |  |  |  |  |  |
| Unmatched | 1.00 | 0.94 |  | 7 |  |  | 0.01 |  |
| Matched | 1.00 | 0.99 |  | 1.7 | 76.1 |  | 0.54 |  |
| Head self-employed |  |  |  |  |  |  |  |  |
| Unmatched | 0.88 | 0.89 |  | -5.5 |  |  | 0.03 |  |
| Matched | 0.88 | 0.88 |  | -0.4 | 93.6 |  | 0.90 |  |
| Rural |  |  |  |  |  |  |  |  |
| Unmatched | 1.92 | 1.95 |  | -15.2 |  |  | <0.001 |  |
| Matched | 1.92 | 1.92 |  | -0.2 | 99 |  | 0.96 |  |
| Household size |  |  |  |  |  |  |  |  |
| Unmatched | 5.96 | 5.85 |  | 3.1 |  |  | 0.23 |  |
| Matched | 5.96 | 5.84 |  | 3.6 | -16.5 |  | 0.17 |  |
| Elderly household member | |  |  |  |  |  |  |  |
| Unmatched | 0.22 | 0.19 |  | 7.9 |  |  | <0.001 |  |
| Matched | 0.22 | 0.21 |  | 2.2 | 72.8 |  | 0.44 |  |
| Expenditure (quintiles) |  |  |  |  |  |  |  |  |
| Unmatched | 2.41 | 2.25 |  | 12.2 |  |  | <0.001 |  |
| Matched | 2.41 | 2.35 |  | 4.7 | 61.7 |  | 0.09 |  |
| Hospital > 1hr |  |  |  |  |  |  |  |  |
| Unmatched | 0.37 | 0.46 |  | -19.3 |  |  | <0.001 |  |
| Matched | 0.37 | 0.37 |  | 1.1 | 94.4 |  | 0.69 |  |
| Radio ownership |  |  |  |  |  |  |  |  |
| Unmatched | 0.70 | 0.64 |  | 13.4 |  |  | <0.001 |  |
| Matched | 0.70 | 0.70 |  | 0.3 | 97.6 |  | 0.91 |  |
| Severity of illness or injury | |  |  |  |  |  |  |  |
| Unmatched | 0.67 | 0.63 |  | 8.1 |  |  | <0.001 |  |
| Matched | 0.67 | 0.67 |  | -0.6 | 92.2 |  | 0.82 |  |
| Disability |  |  |  |  |  |  |  |  |
| Unmatched | 0.04 | 0.03 |  | 4.7 |  |  | 0.06 |  |
| Matched | 0.04 | 0.04 |  | 0 | 100 |  | 1.00 |  |
|  |  |  |  |  |  |  |  |  |
