# Supplementary material for: Inequalities in the benefits of national health insurance on financial protection from out-of-pocket payments and access to health services: cross-sectional evidence from Ghana
Source: Health Policy Plan. 2019 Sep 20;34(9):694–705. doi: 10.1093/heapol/czz093 (PMC6880330; doi:10.1093/heapol/czz093)
Supplement: czz093_Supplementary_Data [file czz093_supplementary_data.zip › czz093-Suppl_data/Supplementary Table 6.docx]

| **Table S6.** Tests of balancing properties for financial risk protection using radius matching, Ghana 2012-2013 | | | | | | | |
| --- | --- | --- | --- | --- | --- | --- | --- |
| Sample | Mean | |  |  | % reduction |  |  |
|  | Insured | Uninsured |  | % bias | bias |  | p>\|t\| |
| Age of head (years) |  |  |  |  |  |  |  |
| Unmatched | 48.93 | 47.86 |  | 7.2 |  |  | <0.001 |
| Matched | 48.88 | 48.99 |  | -0.8 | 89.3 |  | 0.61 |
| Gender of head |  |  |  |  |  |  |  |
| Unmatched | 0.17 | 0.16 |  | 1.4 |  |  | 0.27 |
| Matched | 0.17 | 0.19 |  | -4.9 | -244.2 |  | 0.00 |
| Education of head |  |  |  |  |  |  |  |
| Unmatched | 0.99 | 0.89 |  | 12.2 |  |  | <0.001 |
| Matched | 0.97 | 0.97 |  | 0.3 | 97.2 |  | 0.82 |
| Head self-employed |  |  |  |  |  |  |  |
| Unmatched | 0.88 | 0.91 |  | -9.5 |  |  | <0.001 |
| Matched | 0.90 | 0.89 |  | 2 | 79.5 |  | 0.19 |
| Rural |  |  |  |  |  |  |  |
| Unmatched | 1.92 | 1.96 |  | -15.4 |  |  | <0.001 |
| Matched | 1.95 | 1.94 |  | 3.3 | 78.7 |  | 0.02 |
| Household size |  |  |  |  |  |  |  |
| Unmatched | 6.85 | 6.79 |  | 1.7 |  |  | 0.19 |
| Matched | 6.95 | 6.40 |  | 15.5 | -823 |  | <0.001 |
| Elderly household member | |  |  |  |  |  |  |
| Unmatched | 0.19 | 0.17 |  | 5.8 |  |  | <0.001 |
| Matched | 0.19 | 0.18 |  | 1.7 | 70.9 |  | 0.27 |
| Expenditure (quintiles) |  |  |  |  |  |  |  |
| Unmatched | 2.41 | 2.23 |  | 14.2 |  |  | <0.001 |
| Matched | 2.36 | 2.43 |  | -6.2 | 56.4 |  | <0.001 |
| Hospital > 1hr |  |  |  |  |  |  |  |
| Unmatched | 0.34 | 0.47 |  | -25.9 |  |  | <0.001 |
| Matched | 0.36 | 0.35 |  | 2.1 | 92 |  | 0.16 |
| Radio ownership |  |  |  |  |  |  |  |
| Unmatched | 0.74 | 0.67 |  | 15.2 |  |  | <0.001 |
| Matched | 0.74 | 0.73 |  | 2.1 | 86.3 |  | 0.15 |
| Household member sick or injured | |  |  |  |  |  |  |
| Unmatched | 0.57 | 0.56 |  | 1.5 |  |  | 0.24 |
| Matched | 0.58 | 0.56 |  | 3.4 | -126.9 |  | 0.02 |
| Household member severely sick or injured | | | | | | | |
| Unmatched | 0.43 | 0.42 |  | 2.5 |  |  | 0.05 |
| Matched | 0.43 | 0.42 |  | 3.6 | -43.8 |  | 0.02 |
| Household member with disability | |  |  |  |  |  |  |
| Unmatched | 0.10 | 0.09 |  | 1.5 |  |  | 0.24 |
| Matched | 0.10 | 0.10 |  | 0.4 | 76.6 |  | 0.81 |
|  |  |  |  |  |  |  |  |
|  |  |  |  |  |  |  |  |
