# Supplementary material for: Inequalities in the benefits of national health insurance on financial protection from out-of-pocket payments and access to health services: cross-sectional evidence from Ghana
Source: Health Policy Plan. 2019 Sep 20;34(9):694–705. doi: 10.1093/heapol/czz093 (PMC6880330; doi:10.1093/heapol/czz093)
Supplement: czz093_Supplementary_Data [file czz093_supplementary_data.zip › czz093-Suppl_data/Supplementary Table 7.docx]

| **Table S7.** Tests of balancing properties for financial risk protection using kernel matching, Ghana 2012-2013 | | | | | | | |
| --- | --- | --- | --- | --- | --- | --- | --- |
|  |  |  |  |  |  |  |  |
| Sample | Mean | |  |  | % reduction |  | |
|  | Insured | Uninsured |  | % bias | bias |  | p>\|t\| |
| Age of head (years) |  |  |  |  |  |  |  |
| Unmatched | 48.93 | 47.86 |  | 7.2 |  |  | <0.001 |
| Matched | 48.93 | 48.76 |  | 1.2 | 84 |  | 0.43 |
| Gender of head |  |  |  |  |  |  |  |
| Unmatched | 0.17 | 0.16 |  | 1.4 |  |  | 0.27 |
| Matched | 0.17 | 0.17 |  | -0.1 | 96.4 |  | 0.97 |
| Education of head |  |  |  |  |  |  |  |
| Unmatched | 0.99 | 0.89 |  | 12.2 |  |  | <0.001 |
| Matched | 0.99 | 0.98 |  | 0.9 | 92.8 |  | 0.54 |
| Head self-employed |  |  |  |  |  |  |  |
| Unmatched | 0.88 | 0.91 |  | -9.5 |  |  | <0.001 |
| Matched | 0.88 | 0.89 |  | -2.7 | 72 |  | 0.08 |
| Rural |  |  |  |  |  |  |  |
| Unmatched | 1.92 | 1.96 |  | -15.4 |  |  | <0.001 |
| Matched | 1.92 | 1.93 |  | -3 | 80.5 |  | 0.06 |
| Household size |  |  |  |  |  |  |  |
| Unmatched | 6.85 | 6.79 |  | 1.7 |  |  | 0.19 |
| Matched | 6.85 | 6.85 |  | 0.2 | 88.1 |  | 0.89 |
| Elderly household member | |  |  |  |  |  |  |
| Unmatched | 0.19 | 0.17 |  | 5.8 |  |  | <0.001 |
| Matched | 0.19 | 0.19 |  | 1.2 | 78.8 |  | 0.41 |
| Expenditure (quintiles) |  |  |  |  |  |  |  |
| Unmatched | 2.41 | 2.23 |  | 14.2 |  |  | <0.001 |
| Matched | 2.41 | 2.39 |  | 1.2 | 91.9 |  | 0.43 |
| Hospital > 1hr |  |  |  |  |  |  |  |
| Unmatched | 0.34 | 0.47 |  | -25.9 |  |  | <0.001 |
| Matched | 0.34 | 0.35 |  | -1.3 | 94.9 |  | 0.35 |
| Radio ownership |  |  |  |  |  |  |  |
| Unmatched | 0.74 | 0.67 |  | 15.2 |  |  | <0.001 |
| Matched | 0.74 | 0.74 |  | 0.4 | 97.3 |  | 0.77 |
| Household member sick or injured | |  |  |  |  |  |  |
| Unmatched | 0.57 | 0.56 |  | 1.5 |  |  | 0.24 |
| Matched | 0.57 | 0.57 |  | -0.8 | 48.8 |  | 0.59 |
| Household member severely sick or injured | |  |  |  |  |  |  |
| Unmatched | 0.43 | 0.42 |  | 2.5 |  |  | 0.05 |
| Matched | 0.43 | 0.43 |  | 0.3 | 87.5 |  | 0.83 |
| Household member with disability | |  |  |  |  |  |  |
| Unmatched | 0.10 | 0.09 |  | 1.5 |  |  | 0.24 |
| Matched | 0.10 | 0.09 |  | 1.3 | 12.8 |  | 0.36 |
|  |  |  |  |  |  |  |  |
|  |  |  |  |  |  |  |  |
