# Supplementary material for: Inequalities in the benefits of national health insurance on financial protection from out-of-pocket payments and access to health services: cross-sectional evidence from Ghana
Source: Health Policy Plan. 2019 Sep 20;34(9):694–705. doi: 10.1093/heapol/czz093 (PMC6880330; doi:10.1093/heapol/czz093)
Supplement: czz093_Supplementary_Data [file czz093_supplementary_data.zip › czz093-Suppl_data/Supplementary Table 9.docx]

| **Table S9.** Sensitivity analysis of the propensity score for medical care utilization: effect of ‘calibrated’ confounders, Ghana 2012-2013 | | | | | | | | | | | | |
| --- | --- | --- | --- | --- | --- | --- | --- | --- | --- | --- | --- | --- |
|  | Fraction *U = 1* by treatment / outcome | | | |  | Outcome effect 𝛤 |  | Selection effect 𝛬 |  | ATT |  | 95% CI |
|  | *p11* | *p10* | *p01* | *p00* |  |  |  |  |  |  |  |  |
|  |  |  |  |  |  |  |  |  |  |  |  |  |
| No confounder | 0.00 | 0.00 | 0.00 | 0.00 |  | - |  | - |  | 0.15 |  | (0.12–0.18) |
| Neutral confounder | 0.50 | 0.50 | 0.50 | 0.50 |  | 1.00 |  | 1.00 |  | 0.16 |  | (0.12–0.19) |
|  |  |  |  |  |  |  |  |  |  |  |  |  |
| *Confounder-like* |  |  |  |  |  |  |  |  |  |  |  |  |
| Female | 0.58 | 0.58 | 0.55 | 0.52 |  | 1.13 |  | 1.16 |  | 0.15 |  | (0.11–0.19) |
| Female household head | 0.26 | 0.28 | 0.27 | 0.25 |  | 1.09 |  | 1.02 |  | 0.15 |  | (0.11–0.19) |
| Self-employed household head | 0.80 | 0.85 | 0.80 | 0.83 |  | 0.79 |  | 0.99 |  | 0.15 |  | (0.12–0.19) |
| Rural residence | 0.65 | 0.68 | 0.61 | 0.67 |  | 0.75 |  | 1.12 |  | 0.15 |  | (0.12–0.19) |
| Elderly household member | 0.20 | 0.22 | 0.14 | 0.17 |  | 0.85 |  | 1.44 |  | 0.16 |  | (0.12–0.20) |
| Hospital > 1hr | 0.33 | 0.42 | 0.42 | 0.49 |  | 0.79 |  | 0.67 |  | 0.15 |  | (0.11–0.19) |
| Radio ownership | 0.70 | 0.66 | 0.65 | 0.62 |  | 1.11 |  | 1.26 |  | 0.16 |  | (0.12–0.19) |
| Severity of illness or injury | 0.69 | 0.50 | 0.65 | 0.52 |  | 1.75 |  | 1.22 |  | 0.15 |  | (0.11–0.19) |
| Disability | 0.04 | 0.04 | 0.03 | 0.03 |  | 0.83 |  | 1.34 |  | 0.16 |  | (0.12–0.19) |
|  |  |  |  |  |  |  |  |  |  |  |  |  |
| *Note:* Let *U* be a binary confounding factor whose distribution is fully characterized by the choice of four parameters, $p_{ij}=Pr \left( U=1 \right\vert T=i,Y=j$with $i,j \epsilon\left\{ 0,1 \right\}.$ This gives the probability that *U=1* in each of the four groups defined by treatment and outcome. Given these parameters, we predict a value of *U* for each treated and control subject and re-estimate the ATT by nearest neighbour propensity score matching including the simulated *U* in the set of matching variables *W*. The process is repeated 100 times. 𝛤 denotes the average estimated odds ratio of *U* in the logit model of $\Pr(Y=1\vert T=0,U,W)$. 𝛬 is the average estimated odds ratio of *U* in the logit model of $\Pr(T=1,U,W)$. ATT is the average of the simulated ATTs. CI is the confidence interval. The first row shows the ATT estimate with no confounding factor *U.* The second row shows the ATT estimate with a confounding factor *U* whose outcome and selection effect are insignificant. In each confounder-like row, *U* has been calibrated to match the distribution of the corresponding variable. | | | | | | | | | | | | |
