# Supplementary material for: Inequalities in the benefits of national health insurance on financial protection from out-of-pocket payments and access to health services: cross-sectional evidence from Ghana
Source: Health Policy Plan. 2019 Sep 20;34(9):694–705. doi: 10.1093/heapol/czz093 (PMC6880330; doi:10.1093/heapol/czz093)
Supplement: czz093_Supplementary_Data [file czz093_supplementary_data.zip › czz093-Suppl_data/Supplementary Table 11.docx]

| **Table S11.** Sensitivity analysis of the propensity score for medical care utilization: characterizing ‘killer’ confounders, Ghana 2012-2013 | | | | | | |
| --- | --- | --- | --- | --- | --- | --- |
|  | *s* = 0.1 | *s* = 0.2 | *s* = 0.3 | *s* = 0.4 | *s* = 0.5 | *s* = 0.6 |
|  | 𝛬 ∈ [1.3, 1.4] | 𝛬 ∈ [1.9, 2.2] | 𝛬 ∈ [2.8, 3.6] | 𝛬 ∈ [4.4, 6.7] | 𝛬 ∈ [7.4, 12.3] | 𝛬 ∈ [17.4, 31.0] |
|  |  |  |  |  |  |  |
| *d* = 0.1 𝛤 ∈ [1.5] | 0.15 | 0.14 | 0.13 | 0.12 | 0.11 | 0.11 |
|  | (0.11–0.18) | (0.10–0.18) | (0.09–0.17) | (0.07–0.16) | (0.06–0.16) | (0.05–0.16) |
| *d* = 0.2 𝛤 ∈ [2.3] | 0.14 | 0.12 | 0.10 | 0.08 | 0.06 | 0.06 |
|  | (0.10–0.18) | (0.08–0.16) | (0.06–0.14) | (0.04–0.13) | (0.02–0.11) | (0.01–0.10) |
| *d* = 0.3 𝛤 ∈ [3.6, 3.8] | 0.14 | 0.11 | 0.08 | 0.05 | 0.02 | -0.01 |
|  | (0.10–0.18) | (0.07–0.14) | (0.04–0.12) | (0.004–0.09) | (-0.03–0.06) | (-0.05–0.04) |
| *d* = 0.4 𝛤 ∈ [7.0, 7.1] | 0.13 | 0.09 | 0.05 | 0.01 | -0.03 | -0.07 |
|  | (0.09–0.17) | (0.05–0.13) | (0.01–0.09) | (-0.03–0.05) | (-0.07–0.02) | (-0.11– -0.03) |
| *d* = 0.5 𝛤 ∈ [23.4, 24.3] | 0.13 | 0.07 | 0.02 | -0.03 | -0.09 | -0.14 |
|  | (0.09–0.17) | (0.03–0.11) | (-0.02–0.06) | (-0.07–0.01) | (-0.12– -0.05) | (-0.17– -0.10) |
| *d* = 0.6 𝛤 ∈ [ . ] | 0.13 | 0.06 | 0.003 | -0.06 | -0.12 | -0.18 |
|  | (0.09–0.17) | (0.03–0.10) | (-0.03–0.04) | (-0.09– -0.02) | (-0.15– -0.08) | (-0.21– -0.14) |
|  |  |  |  |  |  |  |
| The differences $d=p_{01}-p_{00}$ and s$=p_{1\cdot}-p_{0\cdot}$ capture the outcome effect of *U* in the absence of treatment and the effect of *U* on the selection into treatment, respectively. *d* and *s* uniquely define the parameters $p_{ij}$, with $i,j \epsilon\left\{ 0,1 \right\}.$ The simulated ATTs associated to the corresponding differences *d* (in rows) and *s* (in columns) are shown in each cell (95% Confidence Intervals in parentheses). Each ATT is averaged over 100 iterations. 𝛤 denotes the average estimated odds ratio of *U* in the logit model of $\Pr(Y=1\vert T=0,U,W)$. 𝛬 is the average estimated odds ratio of *U* in the logit model of $\Pr(T=1,U,W)$. The baseline estimate without confounder is 0.151 (95% CI: 0.12–0.18). | | | | | | |
